# High Incorrect Use of the Standard Error of the Mean (SEM) in Original Articles in Three Cardiovascular Journals Evaluated for 2012

**DOI:** 10.1371/journal.pone.0110364

**Published:** 2014-10-29

**Authors:** Marcel Wullschleger, Soheila Aghlmandi, Marcel Egger, Marcel Zwahlen

**Affiliations:** 1 Department of Physiology, University of Bern, Bern, Switzerland; 2 Institute of Social and Preventive Medicine, University of Bern, Bern, Switzerland; Tokai University, Japan

## Abstract

**Rationale:**

In biomedical journals authors sometimes use the standard error of the mean (SEM) for data description, which has been called inappropriate or incorrect.

**Objective:**

To assess the frequency of incorrect use of SEM in articles in three selected cardiovascular journals.

**Methods and Results:**

All original journal articles published in 2012 in *Cardiovascular Research, Circulation: Heart Failure* and *Circulation Research* were assessed by two assessors for inappropriate use of SEM when providing descriptive information of empirical data. We also assessed whether the authors state in the methods section that the SEM will be used for data description. Of 441 articles included in this survey, 64% (282 articles) contained at least one instance of incorrect use of the SEM, with two journals having a prevalence above 70% and “Circulation: Heart Failure” having the lowest value (27%). In 81% of articles with incorrect use of SEM, the authors had explicitly stated that they use the SEM for data description and in 89% SEM bars were also used instead of 95% confidence intervals. Basic science studies had a 7.4-fold higher level of inappropriate SEM use (74%) than clinical studies (10%).

**Limitations:**

The selection of the three cardiovascular journals was based on a subjective initial impression of observing inappropriate SEM use. The observed results are not representative for all cardiovascular journals.

**Conclusion:**

In three selected cardiovascular journals we found a high level of inappropriate SEM use and explicit methods statements to use it for data description, especially in basic science studies. To improve on this situation, these and other journals should provide clear instructions to authors on how to report descriptive information of empirical data.

## Introduction

In articles of original biomedical research, the authors usually provide descriptive statistical information to illustrate the empirical data they collected. The aim is to describe in a transparent manner the data set as it is without aiming at formal statistical inference. For quantitative measurements (often with clear units of measurement) the information about central tendency (mean or median) and about variability such as standard deviation (SD), range or interquartile range is commonly provided. The SD indicates the dispersion of individual observations about the mean. A low SD indicates less variability while a high SD indicates more spread of the measurements [1]. In describing the variation among observations in the sample, the SD is appropriate in most circumstances [2].

In contrast, inferential statistics makes statements about the values of parameters of the entire population on the basis of the collected data [3]–[5]. Reporting an estimate for the population parameter of interest is often accompanied with a measure of precision. For example, when the population mean is of interest, the sample mean is the estimate and the precision is quantified by providing the 95% confidence interval (CI) of the mean [4], [6]–[9]. To report a 95% CI, instead of a 90% CI or a 67% CI, is only a matter of choice, and has become a convention, intimately related to calling a p-value smaller than 0.05 statistically significant.

To calculate the 95% CI of the mean, one has to use the standard error of the mean (SEM) which is derived from the SD and sample size of the collected data (n) via the formula SEM =  SD/√n [4]. Obviously, the SEM is always smaller than the SD (if more than 1 observation and measurement have been made). The SEM allows for quantifying by how much the sample mean will vary from one sample to the next and by how much the sample mean is different from the true population mean [10]. As the sample SD is an estimate of the variability of individual observations, the SEM is an estimate of the variability of the means of different samples [1]. The SEM is used to compute the 95% CI for a mean, which is done by using the Central Limit Theorem [11] and the formula mean ± reliability coefficient * SEM (e.g., reliability coefficient  = 1.96 taken from the standard normal distribution). The calculated 95% CI contains with 95% probability the true population mean [1]. Displaying a population estimate with the SEM error bar on both sides corresponds to displaying 67% confidence intervals.

In published articles, the SEM is sometimes used to describe the variability of the individual measurements in the collected data [2], [3]. This then gives the impression that the measurements are less variable and more precise [12] (smaller error bar). Using the SEM in order to provide descriptive information on variability of the measurements has been qualified as inappropriate or incorrect [1]–[4], [10]. The frequency of this “statistical error” [4] has amongst others been evaluated in four anesthesia journals in 2001, whereby the prevalence of incorrect use of the SEM was up to 28% of systematically assessed articles [3]. We observed incorrect use of SEM also in some cardiovascular journals. We therefore set out to systematically assess the frequency of incorrect use of SEM in articles published in one calendar year in three selected cardiovascular journals (*Cardiovascular Research, Circulation: Heart Failure* and *Circulation Research*) with one having a more pronounced clinical orientation (*Circulation: Heart Failure*).

## Methods

All original articles (including referenced online supplementary material) published in the year 2012 in *Cardiovascular Research, Circulation: Heart Failure* and *Circulation Research* were systematically assessed on how descriptive statistical information was provided. In 2012 these three journals were among the top 12 ranked journals in the journal group “CARDIAC & CARDIOVASCULAR SYSTEMS” which included 120 journals. “Circulation Research” had a journal impact factor (JIF) of 11.6 and was ranked 4^th^ in this group, “Circulation: Heart Failure” had a JIF of 6.7 and was ranked 7^th^, and “Cardiovascular Research” had a JIF of 5.9 and was ranked 12^th^ (year 2012). We excluded articles without quantitative results, simulation studies, case reports and narrative reviews, as those usually did not report results on original data and measured quantities. Each eligible article was assessed by two independent assessors (MW, SA) for several components on data description, use of the SEM and type of the study (basic science- vs. clinical study; see Extraction Sheet S1). We classified studies as basic science when their main focus was on laboratory methods, mostly involving tissue samples from humans or experimental animals, without a comparative analysis of two or several patient groups. In case of disagreement of the two assessors, a consensus decision was reached with a third person (MZ). For each article we assessed whether there was an explicit statement in the method section stating that data description was done by using "mean (or median) and SD" and/or "mean and SEM" (for example by stating “data will be shown as mean ± SEM”). For the results section including tables and figures we assessed whether we could find an instance of incorrect use of the SEM. Incorrect use could have occurred in two ways: In the first type the SEM was used in tables or figures to describe the variability of the data or measurements without any inferential statistical statement. The second type of incorrect use of SEM was the presentation of results from inferential statistics reflecting situations in which one would have expected 95% CIs in conjunction with p-values from statistical tests of hypotheses. For the analysis we classified the articles as having no, one type only, or both types of inappropriate use of SEM. If the method section stated the use of SD for data description, we assessed whether this was consistent with what was given in the results section, tables or figures. We also recorded when it was unclear throughout the article what type of variability information was provided for data description (“unclear category”). The latter could mean that error bars were included in a figure but neither method section nor the legend of the figure clearly stated what the error bars meant. For the calculation of the frequency of incorrect use of SEM, we referred to the assessed articles (e.g. after exclusion of studies without quantitative results, simulation studies, case reports, narrative reviews). We report our results stratified for the three journals and by type of study (basic science- or clinical study). For each of the main types of inappropriate use of SEM we indicate the frequency, percentage and 95% CI.

## Results

A total of 450 articles were retrieved in these three journals from the year 2012. Of these, 441 qualified to be assessed for incorrect use of SEM (98% of all original articles). Overall, 64% of the selected original articles had instances of inappropriate use of the SEM. The journals “Cardiovascular Research” and “Circulation Research” had a clearly higher level of incorrect use of SEM (72% and 73%) than the third journal “Circulation: Heart Failure” (27%). Overall 6% of the assessed articles had at least one instance of unclear variability information. From the 282 articles (282/441 = 64% of assessed articles; 95% CI: 59–68%) which inappropriately used the SEM, 251 articles (251/441 = 57%; 95% CI: 52–62%) used the SEM for descriptive purposes and also they applied the SEM instead of a 95% CI (e.g. separate figures within the same article). 22 articles (22/441 = 5%; 95% CI: 3–8%) used the SEM exclusively instead of a 95% CI whereas 9 articles (9/441 = 2%; 95% CI: 1–4%) applied SEM in a descriptive manner only. In 81% of articles with incorrect use of SEM, the authors had explicitly stated in the methods section that they intend to use the SEM for data description (see Table 1).

**Table 1:** Characteristics of articles assessed from three cardiovascular journals edited in the year 2012.

Journal	Cardiovascular Research (Oxford Journals), N (%)	Circulation: Heart Failure, N (%)	Circulation Research, N (%)	Total, N (%)
Total number of original articles assessed *	169	85	187	441
Type of study				
Basic science study	159 (94.1)	19 (22.4)	175 (93.6)	353 (80.1)
Clinical study	1 (0.6)	64 (75.3)	3 (1.6)	68 (15.4)
Both basic and clinical study	9 (5.3)	2 (2.4)	9 (4.8)	20 (4.5)
Methods section includes an explicit statement on using SEM for description of the data	109 (64.5)	14 (16.5)	105 (56.2)	228 (51.7)
Methods section includes an explicit statement on using SD for description of the data	34 (20.1)	32 (37.7)	33 (17.7)	99 (22.5)
Unclear throughout the whole article what is used when data is described	6 (3.6)	2 (2.4)	19 (10.2)	27 (6.1)
Use of SEM found in the article				
Inappropriate use of SEM^1^	122 (72.2) [64.8–78.8]	23 (27.1) [18.0–37.8]	137 (73.3) [66.3–79.5]	282 (63.9) [59.3–68.4]
SEM used for descriptive purposes only^1^	3 (1.8) [0.6–5.4]	1 (1.2) [0.2–7.9]	5 (2.7) [1.1–6.3]	9 (2.0) [1.1–3.9]
SEM used instead of 95% CI only^1^	0 (0.0) [0.0–2.2]	7 (8.2) [4.0–16.3]	15 (8.0) [4.9–12.9]	22 (5.0) [3.3–7.5]
Combined use for descriptive purposes and instead of 95% CI^1^	119 (70.4) [63.1–76.8]	15 (17.7) [10.9–27.3]	117 (62.6) [55.4–69.2]	251 (56.9) [52.2–61.5]

*9 studies not assessed (no quantitative results, simulation studies, case reports, narrative reviews).

1 (%) [95% CI (%)].

Of the assessed 441 articles 80% (353/441) presented basic science research, 15% (68/441) clinical research and 5% (20/441) combined both basic science and clinical research. The incorrect use of SEM in studies reporting basic science research was 74% (260/353), more than 7-fold higher than in clinical studies where it was 10% (7/68). Authors of basic science studies stated in 60% of the articles their intention to use the SEM for data description, compared to 4% of authors of clinical studies (see Table 2).

**Table 2:** Results of use of SEM by type of study in three cardiovascular journals (Cardiovascular Research, Circulation: Heart Failure, Circulation Research) edited in the year 2012.

Type of study	Basic science study only, N (%)	Clinical study only, N (%)	Both basic science and clinical study, N (%)	Total, N (%)
Total number of original articles assessed *	353	68	20	441
Methods section includes an explicit statement on using SEM for description of the data	213 (60.3)	3 (4.4)	12 (60.0)	228 (51.7)
Methods section includes an explicit statement on using SD for description of the data	66 (18.7)	30 (44.1)	3 (15.0)	99 (22.5)
Unclear throughout the whole article what is used when data is described	23 (6.5)	3 (4.4)	1 (5.0)	27 (6.1)
Use of SEM found in the article				
Inappropriate use of SEM^1^	260 (73.7) [68.7–78.2]	7 (10.3) [4.2–20.1]	15 (75.0) [50.9–91.3]	282 (64.0) [59.3–68.4]
SEM used for descriptive purposes only^1^	7 (2.0) [1.0–4.1]	1 (1.5) [0.2–9.8]	1 (5.0) [0.7–28.4]	9 (2.0) [1.1–3.9]
SEM used instead of 95% CI only^1^	17 (4.8) [3.0–7.6]	3 (4.4) [1.4–12.9]	2 (10.0) [2.5–32.5]	22 (5.0) [3.3–7.5]
Combined use for descriptive purposes and instead of 95% CI^1^	236 (66.9) [61.8–71.6]	3 (4.4) [1.4–12.9]	12 (60.0) [37.9–78.6]	251 (56.9) [52.2–61.5]

*9 studies not assessed (no quantitative results, simulation studies, case reports, narrative reviews).

1 (%) [95% CI (%)].

## Discussion

This systematic assessment of articles in three cardiovascular journals published in 2012 shows a disturbingly high proportion of articles that use the SEM for data description and inferential statistical statements. Mostly this was accompanied with an explicit methods statement to use the SEM for data description. The level was especially high in basic science studies, i.e. studies focusing on laboratory methods often involving tissue samples from humans or experimental animals. As a consequence of inappropriate use of SEM the reader may assume a smaller variability of the presented original data than actually exists. An incorrectly precise result by edging the outcome with a larger sample size (n), by using SEM instead of SD, may lead to misinterpretation when comparing groups. When making statements about the true parameter of interest, recommendations have been made [1], [9] to provide 95% confidence intervals and, if helpful, p-values for a specific hypothesis of the value of the parameter of interest.

The two journals with the high score of incorrect use of SEM (72% and 73%) each have a proportion of basic science articles over 90%. For the journal with a more clinical orientation of published articles (75% clinical studies), the proportion of incorrect use of the SEM was substantially lower (27%). On the occasion of a similar study reviewing articles published in 2001 in four journals of anesthesiology the incorrect use of SEM was quantified between 12 to 28% of the articles evaluated per journal [3]. Several journals published systematic reviews of statistical methods used when analyzing and reporting data [2], [3], [13]–[18]. The inappropriate use of SEM has been reported for nearly half of the descriptions of data dispersion examined within a review from Avram et al. [13]. Others observed a descriptive statistical error (misuse of SD or SEM) for more than 20% of articles evaluated [14]. MacArthur and Jackson found that 31% of original articles (from Journal of Infectious Diseases, 1982) misused the SEM [17]. Overall we identified 27 articles (27/441 = 6%) with unclear description of a shown measure of dispersion. This figure is rather low when comparing with other manuscripts (e.g. Olsen, 2003: 14% [2]; Felson, 1984: 13% to 9%[16]). Only Nagele (2003: 2%) [3] reported a lower portion. Although some articles assessed within our study used the SEM instead of a 95% CI (which may be qualified as a "minor misuse of SEM"), the majority of articles with inappropriate use of SEM contained at least some table or figure using SEM for descriptive purposes.

Among the three selected cardiovascular journals, two had a high proportion of basic science- or combined (basic science and clinical) studies. Both of these two journals showed a high level of inappropriate use of SEM. Whether this can fully explain our findings is unclear, as other reviews [2], [3], [13]–[18] also included relevant proportions of basic science or laboratory articles. Nagele stated that laboratory reports and clinical studies were equally affected, except for one out of four journals where 90% of studies with incorrect SEM use were in basic science studies [3]. One might speculate about the influence of the underlying institutions or societies associated with certain journals or disease domains. Previous reviews covered areas that are predominantly of clinical nature (e.g. Nagele, 2003 [3]: anaesthesia; Cruess, 1989 [14]: tropical medicine, hygiene; MacArthur, 1984 [17]: infectious diseases; Felson, 1984 [16]: arthritis, rheumatism). Comprehensive journals e.g. in cardiovascular science (or neuroscience) publish laboratory studies conducted in clinical as well as in pre-clinical research institutions. It might be that the strategies recommended for data description and statistical analysis are different in pre-clinical institutions and in institutions that also include clinical service which are also involved in clinical studies and trials for which reporting standards have been established over the last decades (http://www.consort-statement.org/). However we note as limitations that we could not assess whether the institution of the corresponding author also involved clinical service, and we did not randomly (but subjectively) select the three cardiovascular journals for systematic assessment. Therefore, our results do not necessarily reflect the situation of the whole group of cardiovascular journals.

## Conclusions

The SEM is still widely and inappropriately used in articles published in three selected journals specialized in cardiovascular research ranked among the top 12 of 120 journals listed in “cardiac & cardiovascular systems”. This is often accompanied by an explicit methods statement about the use of the SEM for data description, especially in basic science studies. None of the journals examined provided explicit statistical guidelines for authors and/or reviewers. Explicit journal policies could help to improve descriptive statistics in their articles by adapting their recommendations and checklists for conducting peer reviews of the submitted articles. The standard method to describe the original data collected in a biomedical study should be to provide mean and SD, or median and quantile information. Bar graphs with means and “error bars” should be avoided and box plots used more often. Journals should give authors clearer instructions on how to prepare their figures. Furthermore, the review process should help to reduce the level of incorrect use of SEM, which can lead to unclear data presentation and misinterpretation of results.

## Supporting Information

Extraction Sheet S1 **Provides the extraction sheet used.**
(DOC)

PRISMA Checklist S1 **Provides the PRISMA Checklist for this review.**
(DOC)

PRISMA Flowchart S1 **Provides the PRISMA Flowchart for this review.**
(DOC)

Data S1 **Provides the extracted data.**
(XLS)
