# Supplementary material for: High Incorrect Use of the Standard Error of the Mean (SEM) in Original Articles in Three Cardiovascular Journals Evaluated for 2012
Source: PLoS One. 2014 Oct 29;9(10):e110364. doi: 10.1371/journal.pone.0110364 (PMC4212967; doi:10.1371/journal.pone.0110364)
Supplement: Extraction Sheet S1 — Provides the extraction sheet used. (DOC) [file pone.0110364.s001.doc]

Supplementary Material

**Extraction sheet**

|  | Assessor | Date of assessment: : __________________________ __________________________ |
| --- | --- | --- |
|  | First author |  |
|  | Year of publication | 2012 |
|  | Journal | ❒ *Cardiovascular Research*  ❒ *Circulation Heart Failure*  ❒  *Circulation Research* |
|  | Volume |  |
|  | First page of publication |  |
|  | Exclusion of the whole article from assessment of misuse due to | ❒ No quantitative results  ❒ Case report  ❒ Narrative review  ❒ Other ____________________________________ |
|  | Type of study | This is a basic science or laboratory study  ❒ Yes ❒ No ❒ Unclear  This is a clinical study  ❒ Yes ❒ No ❒ Unclear  Comments : ____________________________________ |
|  | Statements about the statistical methods used in the article | There is **an explicit statement** in methods section stating that data are shown (or presented) as mean (or median) ± **standard error of the mean (or SEM)**  ❒ Yes ❒ No ❒ Unclear  There is **an explicit statement** in methods section stating that data are shown (or presented) as mean (or median) ± **standard deviation (or SD)**  ❒ Yes ❒ No ❒ Unclear  There is **no explicit** **statement anywhere in the article** about what is shown in tables and figures (should be rare)  ❒ Yes ❒ No ❒ Unclear |
|  | Now questions about misuse of the SEM anywhere in the article | Was there **use of SEM** **to summarize data** anywhere in the article?  ❒ Yes ❒ No ❒ Unclear  Indicate table where this type of misuse can be seen:  Table Nr:  Indicate figure where misuse can be seen:  Figure Nr: |
|  |  | Was there **use of SEM instead of 95% CI** in the article? (typically when also showing whether differences were statistically significant)  ❒ Yes ❒ No ❒ Unclear  Indicate table where this type of misuse can be seen:  Table Nr:  Indicate figure where misuse can be seen:  Figure Nr: |
|  |  |  |
