# Supplementary material for: High Incorrect Use of the Standard Error of the Mean (SEM) in Original Articles in Three Cardiovascular Journals Evaluated for 2012
Source: PLoS One. 2014 Oct 29;9(10):e110364. doi: 10.1371/journal.pone.0110364 (PMC4212967; doi:10.1371/journal.pone.0110364)
Supplement: PRISMA Flowchart S1 — Provides the PRISMA Flowchart for this review. (DOC) [file pone.0110364.s003.doc]

**Screening**

**Included**

**Eligibility**

**Identification**

Records identified through database searching
(n = 450)

Additional records identified through other sources
(n = 0)

Records after duplicates removed
(n = 450)

Records screened
(n = 450)

Records excluded
(n = 0)

Full-text articles assessed for eligibility
(n = 450)

Full-text articles excluded, with reasons
(n = 9)

Studies included in qualitative synthesis
(n = 441)

Studies included in quantitative synthesis (meta-analysis)
(n = 441)
